# The mediating role of psychological capital between chameleon leadership and group cohesion among staff nurses: a cross-sectional path analysis study

**DOI:** 10.1186/s12912-025-03667-9

**Published:** 2025-08-05

**Authors:** Aida Mahmoud Abdel-Azeem, Ayman Mohamed El-Ashry, Safa Mohamed Amin, Fatma Fouad Elsayed

**Affiliations:** 1 Nursing administration,Faculty of Nursing, Beni-Suef University, Beni-Suef, Egypt; 2 Psychiatric and Mental Health Nursing, Faculty of Nursing, Alexandria University, Alexandria, Egypt; 3 Psychiatric and Mental Health Nursing, Faculty of Nursing, Beni-Suef University, Beni-Suef, Egypt; 4 Nursing Administration, Faculty of Nursing, Beni-Suef University, Beni-Suef, Egypt

**Keywords:** Chameleon leadership, Group cohesion, Psychological capital, Staff nurses

## Abstract

**Background:**

Healthcare organizations are increasingly challenged by crises, workforce shortages, and technological changes that require adaptable leadership styles. Chameleon leadership is style of leadership that adapts quickly and consistently in response to environmental changes and possesses the capability to mirror the strategies of rival organizations. It characterized by flexibility and emotional adaptability alongside the psychological capital (PsyCap) of nursing staff, may influence group cohesion, a crucial factor for effective teamwork and patient care.

**Aim:**

To examine the mediating role of psychological capital between chameleon leadership and group cohesion among staff nurses.

**Design and method:**

A cross-sectional, correlational descriptive study was conducted with a convenience sample of 282 staff nurses at Beni-Suef University Hospital. Data were collected using an online survey consisting of sociodemographic details, the Psychological Capital Questionnaire (PCQ), the Chameleon Leadership Scale, and the Group Cohesion Scale. Statistical analyses included Pearson’s correlation, multiple regression, and path analyses.

**Results:**

Staff nurses reported moderate levels of chameleon leadership (62.87%) and psychological capital (71.65%), but low group cohesion (26.66%). Significant positive correlations were found between chameleon leadership, PsyCap, and group cohesion (*p* < 0.001). Path analysis showed that psychological capital (especially resilience and self-efficacy) significantly mediates the relationship between chameleon leadership and group cohesion. The model explains 54.1% of the variance in group cohesion.

**Conclusions:**

There were moderate associations between chameleon leadership and psychological capital and group cohesion of nurses, with a mediating effect on psychological capital among the two variables.

**Implications for nursing and health policy:**

Leadership development programs should focus on enhancing nurse leaders’ adaptive behaviors and psychological capital to foster stronger team cohesion. Hospital administrations should also integrate psychological capital building initiatives into staff development strategies to improve work environments and patient care outcomes.

**Clinical trial number:**

Not applicable.

## Background

In the contemporary healthcare landscape, delivering safe, efficient, and high-quality care is not optional—it is a strategic imperative. Healthcare organizations worldwide face mounting pressures from persistent crises, workforce shortages, and rapid technological changes [1]. These challenges are further exacerbated in politically unstable regions like Egypt, where economic disruptions, infrastructure limitations, and staffing volatility add further complexity to healthcare delivery [2, 3]. Amid these turbulent conditions, hospital administrators and nurse leaders must adopt leadership styles that emphasize flexibility, innovation, and speed to effectively navigate the demands of modern health systems [1].

Leadership, often viewed as the cornerstone of organizational performance, is particularly influential in shaping work environments in complex and high-pressure settings such as hospitals [4]. Traditional, rigid leadership styles have proven inadequate in such dynamic contexts, often resulting in communication breakdowns, low morale, and compromised patient outcomes [5]. As a response, newer, adaptive models of leadership have gained traction, especially Chameleon Leadership, which emphasizes behavioral flexibility, situational responsiveness, and emotional intelligence [6, 7].

Chameleon Leadership is characterized by a leader’s ability to strategically adapt their behavior, tone, communication style, and emotional responses according to situational demands—without compromising their core identity [6]. Unlike fixed leadership paradigms, chameleon leaders employ empathy, diplomacy, and flexibility as tools to manage both internal team dynamics and external organizational pressures [7]. This approach enables leaders to “blend in” when necessary, protect their teams from destabilizing forces, and maintain performance even under volatile conditions—qualities especially critical in high-stakes environments like healthcare [7].

In parallel, Psychological Capital (PsyCap) has emerged as a vital internal resource among healthcare workers, particularly nurses. Defined by Luthans et al. as a positive psychological state of development composed of four dimensions**—**hope, efficacy, resilience, and optimism (HERO)—PsyCap reflects individuals’ capacity to persevere, recover from setbacks, and envision positive outcomes [8].

Each element of PsyCap plays a distinct yet interconnected role: Hope represents goal-directed energy and the capacity to find multiple pathways to success. Self-efficacy reflects confidence in one’s ability to perform tasks. Resilience indicates one’s capacity to bounce back from adversity. Optimism relates to expecting positive outcomes in the face of uncertainty [9, 10].

Research has consistently shown that high PsyCap is associated with greater work engagement, job satisfaction, stress reduction, and lower burnout among nurses [11]. As such, strengthening PsyCap is increasingly recognized as a strategy for enhancing both employee well-being and healthcare outcomes.

According to social learning theory, employees tend to model the behaviors and emotional states of their leaders [12]. Thus, when leaders exhibit high levels of psychological capital—resilience, optimism, self-confidence—they influence their teams by fostering similar positive psychological states. This “emotional contagion” mechanism enhances not only individual well-being but also group-level dynamics such as trust, collaboration, and communication [12].

This brings into focus the critical role of Group Cohesion—defined as the emotional bonding, mutual trust, and shared commitment among team members [13]. In nursing units, strong group cohesion has been linked to improved communication, lower conflict, higher satisfaction, and ultimately, better patient care [14]. Cohesive teams also demonstrate superior performance and resilience during crises [15, 16]. Importantly, emotionally intelligent and supportive leadership has been identified as a major determinant of team cohesion [17].

However, while the individual relationships between leadership style, PsyCap, and group cohesion are well documented, there is limited empirical research on how these variables interact. Particularly, the mediating role of PsyCap in the relationship between adaptive leadership styles like chameleon leadership and group cohesion remains underexplored [18, 19].

Moreover, in high-stakes clinical environments, such as emergency rooms, intensive care units, and understaffed wards, nurses are often required to make rapid clinical decisions, coordinate care across disciplines, and manage emotional stress from patients and families. Leaders who embody chameleon traits can modulate their behavior to stabilize teams, de-escalate conflicts, and respond to evolving crises functions critical to sustaining operational continuity and morale. Simultaneously, nurses with high psychological capital—especially resilience and self-efficacy—are better able to maintain clinical accuracy under fatigue, engage in collaborative care, and bounce back from emotionally taxing shifts. Thus, the interplay between adaptive leadership and psychological resilience directly informs the capacity of nursing teams to function safely and effectively under pressure [16, 17].

### Problem statement

There is a clear gap in the literature regarding how chameleon leadership behaviors influence team dynamics through psychological mechanisms. Specifically, the majority of research has focused on transformational or authentic leadership models, with little attention given to adaptive leadership models, such as Chameleon Leadership, which may be better suited to healthcare’s volatile, uncertain environments [7, 20]. Given the persistent challenges faced by healthcare systems, especially in politically unstable regions such as Egypt, it is crucial to identify leadership strategies that not only adapt to change but also empower nurses psychologically and enhance group cohesion [21]. This study aims to address this critical research gap by examining the direct and indirect effects of chameleon leadership on group cohesion, with PsyCap as a mediating variable, among staff nurses in an Egyptian teaching hospital.

### Significance of the study

The significance of this study lies in its timely focus on adaptive leadership, psychological resilience, and team unity in one of the world’s most demanding professions. First, by introducing and operationalizing the concept of chameleon leadership within a nursing context, this study offers a novel perspective that aligns with the realities of modern healthcare. Second, it highlights the transformational potential of psychological capital as a modifiable trait that organizations can develop through training, mentorship, and support systems.

Third, the findings can directly inform leadership development programs and hospital policies aimed at enhancing team cohesion, reducing burnout, and fostering a more supportive work climate. Furthermore, the implications extend to healthcare policymakers who seek to establish accreditation standards that include psychological safety and leadership adaptability as indicators of excellence [14, 21].

### Aim of the study

This study aims to:

- Explore the relationship between chameleon leadership, psychological capital, and group cohesion among staff nurses.
- Investigate whether psychological capital mediates the relationship between chameleon leadership and group cohesion.

### Research hypotheses?

#### H1

Chameleon leadership is positively associated with staff nurses’ psychological capital.

#### H2

Psychological capital is positively associated with group cohesion among staff nurses.

#### H3

Psychological capital mediates the relationship between chameleon leadership and group cohesion.

### Research design and setting

This study employed a cross-sectional, correlational descriptive design and was conducted following Strengthening the Reporting of Observational Studies in Epidemiology (STROBE) guidelines at Beni-Suef University Hospital. This hospital was chosen due to different reasons, such as: The type of sample (convenience), it was restricted by the ethical committee, and also it represents the largest hospital in Beni-Suef governorate.

The hospital, a major teaching institution with a capacity of 483 beds, is one of the largest healthcare facilities in the Beni Suef Governorate, serving a wide and diverse population across the region. The hospital was housed within a six-story building, with each floor designated for specific medical services and units. The first floor accommodates essential services, such as the emergency department, hemodialysis unit, surgical intensive care unit, laundry, kitchen, and sterilization unit. The second floor includes the oncology unit, radiology department, orthopedic unit, laboratory services, and various outpatient clinics. The third floor features the general intensive care unit along with the operating rooms, which are subdivided into general and specialized surgical areas. The fourth floor is dedicated to surgical wards and includes resting rooms for the physicians. On the fifth floor, the cardiac, medical, and pediatric departments were located. Finally, the sixth floor houses the obstetrics department, the ear, nose, and throat (E.N.T) unit, and an endemic disease unit.

### Sample size calculation and sampling

The sample size for this study was calculated using the Epi Info-7 program. With a total population of 952 staff nurses, an expected frequency of 50%, a confidence limit of 5%, and a confidence level of 95%, the program estimated the minimum required sample size of 272 participants. For this study, a convenience sample of 282 staff nurses from various departments within a specified setting was selected.

### Inclusion and exclusion criteria

Eligible participants were required to meet the following inclusion criteria: (a) hold certification and a valid nursing license and (b) have at least six months of work experience in their current unit(c) both genders. Nurses who were undergoing training or on leave during the data collection period were excluded from participation.

### Measurements

The data collection involved four measurement tools.

#### Tool 1: Socio-demographic data

This section captures information such as age, marital status, number of children, place of residence, educational background, department, academic rank, and sex.

#### Tool 2: psychological capital questionnaire

It was originally developed by Luthans et al. (2007) to assess an individual’s positive psychological state, commonly referred to as PsyCap [8]. The scale consists of 24 items divided equally across four dimensions: self-efficacy, hope, resilience, and optimism. Each construct represents a distinct psychological capability-efficacy reflects one’s confidence in handling challenging tasks; hope relates to goal-directed energy and the ability to devise pathways to achieve objectives; resilience captures the ability to recover from setbacks; and optimism denotes a positive outlook about future success. Respondents rated each item on a 3-point Likert scale (1 = disagree, 2 = neutral, 3 = agree). The total score ranges from 24 to 72, with higher scores indicating stronger psychological capital. For interpretative clarity, scores between 24 and 36 were categorized as low, 37–55 as moderate, and 56–72 as high. In the original validation by Luthans et al., the PCQ demonstrated high internal consistency (Cronbach’s α = 0.88), while in the current study, it yielded a reliability coefficient of 0.79. Permission to use the instrument was obtained from MindGarden Inc. (https://www.mindgarden.com*).*

#### Tool 3: the arabic version of chameleon leadership scale

The third tool was the Arabic Version of the Chameleon Leadership Scale, adapted and validated by Alsaadawi (2023) [22]. This 10-item instrument measures employees’ perceptions of chameleon leadership behavior specifically, a leader’s ability to adapt their behavior, tone, and emotional responses to different organizational contexts while maintaining their core values. Items such as “My supervisor adjusts their behavior depending on who they are interacting with” reflect this behavioral flexibility and situational awareness. Responses were recorded on a 5-point Likert scale ranging from 1 (strongly disagree) to 5 (strongly agree), with higher scores indicating greater perceived chameleon leadership. The original study reported a Cronbach’s alpha of 0.89, while the current study found a similarly high reliability coefficient of 0.87, indicating strong internal consistency.

#### Tool 4: group cohesion scale

The fourth instrument was the Group Cohesion Scale–Revised (GCS-R), developed by Treadwell et al. (2001), which assesses the degree of emotional bonding, trust, and collaborative engagement among team members [15]. The scale includes 24 items across three subdomains: cohesion to the group, cohesion to the leader, and member satisfaction. Unlike the previous version mistakenly described, the revised scale utilizes a 5-point Likert format (1 = strongly disagree to 5 = strongly agree). Higher scores reflect stronger group cohesion. The total score ranges from 24 to 120, with scores of 24–55 interpreted as low cohesion, 56–87 as moderate, and 88–120 as high. The original version demonstrated high internal reliability, with Cronbach’s alpha values ranging from 0.84 to 0.91 depending on the subscale. In the present study, the overall Cronbach’s alpha for the scale was 0.91, indicating excellent internal consistency.

### Ethical consideration

#### Ethical approval

for this study was obtained from the Research Ethics Committee at the Faculty of Medicine, Beni-Suef University, Egypt. The approval number is: IRB**/**FMBSUREC/03122024/ABD-AZEEM. The study was conducted in full accordance with the ethical principles outlined in the Declaration of Helsinki and its later amendments. Prior to data collection, informed consent was obtained electronically from all participants via an online consent form embedded in the survey. Participation was voluntary, and respondents were assured of their right to withdraw at any time without penalty. Anonymity and confidentiality of the data were strictly maintained, and no identifying information was collected or reported.

### Tools validity

The validity of the study instruments was assessed through face and content validation by a panel of three experts specializing in Nursing Administration and Psychiatric/Mental Health Nursing. These experts were selected based on their academic experience and familiarity with leadership, psychological constructs, and psychometric evaluation.

Each expert independently reviewed the Arabic-translated versions of the Psychological Capital Questionnaire (PCQ) and the Group Cohesion Scale to evaluate their linguistic clarity, cultural appropriateness, relevance to the nursing context, and the conceptual alignment of each item with the intended constructs.

Following their individual assessments, a consensus meeting was held, during which feedback was consolidated, discrepancies were discussed, and agreed-upon modifications were made to ensure semantic and contextual consistency. Items were retained, reworded, or refined based on mutual agreement. No items were eliminated, as the panel unanimously found the content to be representative and comprehensive after minor linguistic adjustments. This validation process enhanced the content validity and cultural adaptation of the instruments, ensuring their appropriateness for use in the Egyptian nursing context.

For the PCQ, exploratory factor analysis (EFA) was performed after translation. The initial factor loadings ranged between 0.515 and 0.856, and after applying varimax rotation, the loadings improved from 0.710 to 0.956, exceeding the minimum acceptable threshold of 0.34. These factors account for 79.506% of the total variance.

The Group Cohesion Scale followed the same rigorous validation procedures. After translation, an exploratory factor analysis was conducted. The initial factor loadings ranged from 0.498 to 0.801, which improved to between 0.672 and 0.912 after varimax rotation. This structure accounts for 76.814% of the total variance. The KMO measure was 0.936, indicating an excellent sampling adequacy. Bartlett’s test of sphericity showed a highly significant result (*p* ≤ 0.001). No items were eliminated, thus confirming the robustness of the translated scale.

### Pilot study

A pilot study was conducted with a sample of 30 staff nurses (approximately 10% of the total sample) from Beni-Suef University Hospital. The purpose of the pilot study was to evaluate the clarity, cultural relevance, and feasibility of the research instruments, as well as to estimate the average time required for completion. Participants reported that the items were generally clear and understandable, and the completion time ranged between 30 and 45 min. No major issues or conceptual misunderstandings were identified. However, based on participant feedback, minor linguistic refinements were made to a few items in the Arabic translations to improve phrasing and ensure semantic precision. These adjustments were approved by the expert panel prior to full-scale data collection. The data from the pilot study were not included in the final analysis.

### Data collection

This study was conducted following the official approval of the Faculty of Nursing at Beni-Suef University. Data collection was facilitated through a web-based survey created on Google Forms. The survey link was shared via WhatsApp with approximately 450 staff nurses across all units, as facilitated by nursing students during their clinical placements. Of these, 282 completed responses were received, yielding a response rate of approximately 62.7%an acceptable rate for online surveys in healthcare settings. The one-month data collection period (October 15 to November 15, 2024) was strategically selected to align with both ethical approval timelines and academic rotations, ensuring that participation occurred during a relatively stable period with minimal disruption to clinical operations. This timeframe also avoided major holidays and peak patient load periods, optimizing nurses’ availability to engage with the survey.

To support distribution and maximize reach, nursing students who were undergoing hospital-based training volunteered to assist by sharing the survey link in official departmental WhatsApp groups. These students were not part of the formal research team and had no access to the data collected. Their role was limited to logistical assistance in disseminating the questionnaire link. Prior to participation, nurses were required to provide informed consent by clicking the “I agree” button on an online consent form before accessing the survey questions. To ensure completeness, all questions were mandatory, preventing the submission of partial responses. Once the target sample size was achieved, the survey was completed.

### Statistical analysis

Data were entered and analyzed using IBM SPSS software version 26.0. Pearson’s correlation coefficient was used to examine the relationships between normally distributed quantitative variables. A multiple regression analysis was conducted to identify the factors influencing group cohesion. Additionally, a path analysis was performed to explore both the direct and indirect effects of chameleon leadership on group cohesion, with psychological capital acting as a mediator. Path analysis was performed using SPSS Analysis of Moment Structures (AMOS) version 26. Model fit was evaluated based on several criteria, including the Root Mean Square Error of Approximation (RMSEA ≤ 0.10), Comparative Fit Index and Incremental Fit Index (CFI and IFI > 0.90, preferably ≥ 0.95). Statistical significance was set at the 5% level.

In statistics, correlation generally refers to the measure of the linear relationship between two quantitative variables, whether the association is causal. Multiple linear regression, a statistical technique used in this study, models the linear relationship between a dependent variable and one or more independent variables, estimating unknown parameters using observed data. Linear regression assumes a straight-line relationship between the predictors and the outcome variable.

## Results

Table 1 presents the demographic characteristics. The staff nurses primarily comprised younger individuals (86.6% under 34 years of age). Females dominated (77%) and most were married (55%). Educationally, 77.3% held a bachelor’s degree in nursing and 56.4% had less than five years of experience.

Table 2 presents the descriptive statistics of the study variables, including both raw and percentage-based mean scores. The percentage scores represent the participants’ average responses relative to the maximum possible score for each respective scale, calculated to facilitate comparison across instruments with different scoring ranges. The findings show that staff nurses perceived a moderate level of chameleon leadership (62.87% ± 18.86), suggesting a fair degree of leadership adaptability in their work environment. Psychological capital also scored moderately high overall (71.65% ± 14.12), with work self-efficacy (72.03%) and resilience (70.67%) ranking highest among its dimensions. However, hope was notably low (24.29%), indicating limited confidence in goal-directed energy and future-oriented motivation. Optimism scored slightly below the overall PsyCap average (67.70%). Alarmingly, group cohesion scored very low (26.66% ± 6.84), highlighting deficiencies in teamwork, mutual trust, and interpersonal unity among nursing staff. These results emphasize the need to enhance adaptive leadership behaviors and psychological resources—particularly hope and team cohesion—to improve staff well-being and care delivery in high-pressure clinical settings.

Table 3 presents the correlations among the study variables. All variables were significantly correlated (*p* < 0.001). Chameleon Leadership showed moderate associations with PsyCap (*r* = 0.535) and Group Cohesion (*r* = 0.511). PsyCap components (resilience, *r* = 0.879) and Group Cohesion (*r* = 0.726) exhibited strong interrelations, highlighting their interconnectedness. Hope, despite its low mean, correlated robustly with other variables (*r* = 0.684 with Work Self-Efficacy), underscoring its role in the network of psychological resources.

Table 4 shows the regression analysis effect of Chameleon Leadership and Psychological capital on Group Cohesion among staff nurses. The model explained 54.1% of the variance in Group Cohesion (*R²* = 0.556). Chameleon Leadership (β = 0.169, *p* = 0.001), Work Self-Efficacy (β = 0.202, *p* = 0.001), Hope (β = 0.187, *p* = 0.002), and Resilience (β = 0.225, *p* = 0.002) were significant predictors. Optimism approached significance (β = 0.132, *p* = 0.051), suggesting marginal influence. Leadership and psychological resources collectively drive cohesion, with resilience being the strongest contributor to cohesion.

**Fig. 1:**
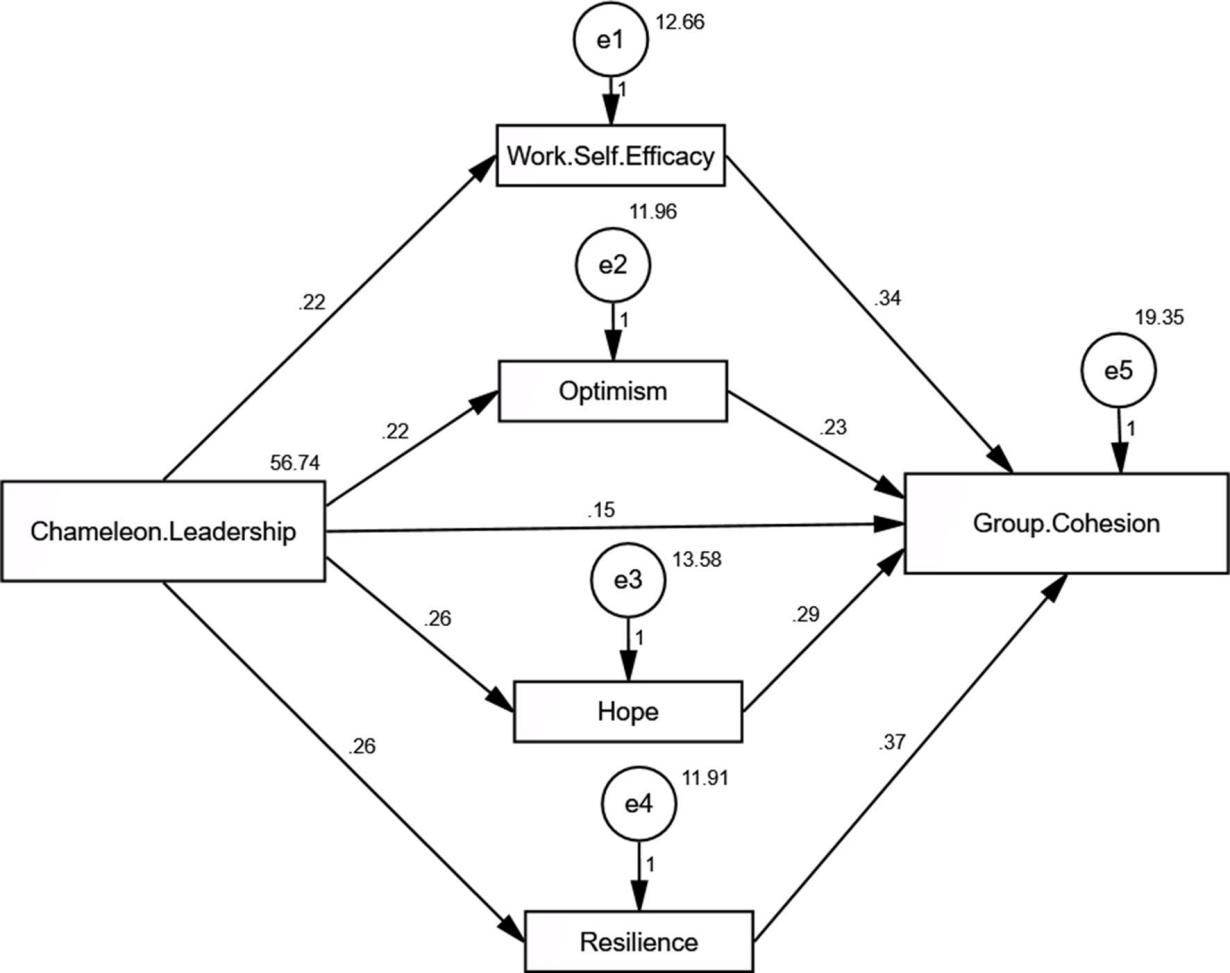
Path analysis to detect the direct and indirect effect of chameleon leadership scale on group cohesion scale: the mediating role of psychological capital (*n* = 282)

Table 5 Fig. 1 present the direct and indirect effects of Chameleon Leadership on various psychological and group dynamics variables, focusing on the mediating effects of Work Self-Efficacy, Optimism, Hope, and Resilience. Chameleon Leadership had a significant direct effect on Work Self-Efficacy (0.220, *p* < 0.001), optimism (0.217, *p* < 0.001), hope (0.260, *p* < 0.001), and resilience (0.264, *p* < 0.001), as indicated by the high critical ratio (C.R.) values and statistically significant p-values. These results suggest that Chameleon Leadership strongly influences these psychological traits. When examining Group Cohesion, Chameleon Leadership had both a direct effect (0.147, *p* < 0.001) and an indirect effect (0.097, *p* < 0.001), mediated by Work Self-Efficacy, Optimism, Hope, and Resilience. Specifically, Work Self-Efficacy (0.338, *p* < 0.001), optimism (0.225, *p* = 0.048), hope (0.293, *p* = 0.002), and resilience (0.371, *p* = 0.002) significantly contributed to Group Cohesion, highlighting their mediating roles. Resilience had the strongest mediating effect on Group Cohesion, followed by Work Self-Efficacy, Hope, and Optimism. The model fit parameters (CFI = 1.000, IFI = 1.000, RMSEA = 0.089) indicated an excellent fit for the data, although the RMSEA value was slightly above the ideal threshold of 0.08, suggesting a minor room for improvement.

## Discussion

Leaders who exhibit chameleon-like qualities possess a valuable degree of adaptability, emotional intelligence, and contextual awareness that allows them to operate effectively across diverse work environments. They adjust seamlessly to evolving demands and interpersonal dynamics while fostering inclusive, psychologically safe settings that support cohesive, high-functioning teams. Although their outward behavior is flexible, these leaders maintain a stable core identity and values, contributing to relational trust and consistency in leadership practice [23].

Similarly, individuals with high levels of psychological capital (PsyCap) tend to demonstrate stronger self-confidence, resilience, and perseverance in pursuing work-related goals. These individuals are often more capable of learning from setbacks, maintaining constructive attitudes, and sustaining motivation under pressure [24]. In healthcare environments—particularly nursing—such psychological resources are essential for managing occupational stress and maintaining collaborative team functioning.

Findings from this study revealed that approximately two-thirds of the participating nurses perceived their leaders to exhibit a moderate level of chameleon leadership. In parallel, most nurses reported moderate psychological capital. These patterns may be linked to the nature of nursing leadership in culturally diverse and high-pressure settings, where leaders must frequently navigate interpersonal challenges, conflict, and resource limitations [16, 19]. The observed levels of PsyCap may also reflect the psychological influence of adaptive leaders who foster motivation and emotional support through context-sensitive leadership behavior.

These findings are consistent with prior research. For instance, Durrah and Kahwaji (2022) reported moderate expressions of chameleon leadership in healthcare contexts [19], and Abd Alhosine and Mahmood (2022) found similar adoption patterns among academic leaders [25]. Likewise, moderate PsyCap levels have been documented among Chinese and Indonesian nurses (Song & Landicho, 2023; Syam & Arifin, 2021) [26, 27], although Jafarizadeh et al. (2019) observed higher levels in psychiatric settings, possibly due to different organizational dynamics [28].

When examining the specific dimensions of PsyCap, our results showed that self-efficacy and resilience scored relatively higher, while optimism was lower and hope notably low. These findings may reflect broader contextual stressors, such as economic uncertainty and healthcare system strain in Egypt, which can affect goal-oriented motivation and expectations about the future. Additionally, the low group cohesion reported by nurses may be a function of inter-team stress, understaffing, and heavy workloads, which can impair collaborative trust and communication.

These trends partially align with prior studies. Song and Landicho (2023) found self-efficacy to be the highest-rated PsyCap dimension, although their respondents reported higher hope and optimism than in our sample [26]. In contrast, studies such as Cruz et al. (2018) among Filipino nurses found above-average optimism levels [29], suggesting that national context and organizational support may influence the development of PsyCap dimensions. Similarly, Wang et al. (2022) reported moderate group cohesion among psychiatric nurses in China [14], indicating variability based on healthcare setting and specialty.

The current findings demonstrated a moderate positive association between chameleon leadership, psychological capital, and group cohesion. These relationships suggest that when leaders adapt empathetically to team needs and environmental stressors, they may help foster psychological strengths among their team members. In turn, enhanced PsyCap may contribute to stronger interpersonal trust and team integration. These interpretations align with Williams (2022), who emphasized that adaptive leaders create emotionally positive environments that support workplace cohesion [18], and with Massoudi (2022), who linked chameleon leadership to improved organizational performance [7].

Abbas (2025) also emphasized that chameleon leadership can influence employees’ psychological maturity by shifting internal control perceptions, which may indirectly affect collaborative outcomes [30]. Our findings suggest that chameleon leadership behaviors are positively associated with PsyCap, a result that resonates with the emotional contagion model in leadership theory, whereby the leader’s psychological state influences that of the followers [12].

Moreover, the data revealed a positive association between psychological capital and group cohesion, in line with Cesaro (2016), who reported that team cohesion was significantly related to employees’ PsyCap levels [16]. This connection highlights the role of PsyCap not only as an individual resource but also as a contributor to collective dynamics within teams. Similar conclusions were drawn by El (2019), who emphasized the interactional origins of psychological resources in workplace settings [31].

Finally, the study’s path analysis supported the mediating role of psychological capital in the relationship between chameleon leadership and group cohesion. That is, the relationship between leadership adaptability and team unity appears to operate—at least partially—through the enhancement of psychological resources such as resilience, hope, and self-efficacy. These findings are consistent with Cesaro (2016), who found that team cohesion can be strengthened when leaders foster positive psychological states among staff [16]. However, this result contrasts with Wu et al. (2022), who reported a negative moderating effect of psychological capital between leadership and organizational climate in a sample of educators and athletes [32]. Such discrepancies may be attributed to differences in professional context, role expectations, and the nature of teamwork involved [33, 34].

To our knowledge, this study is the first to empirically explore the interplay between chameleon leadership, psychological capital, and group cohesion among staff nurses. While the cross-sectional design limits our ability to infer causation, the findings contribute to a growing understanding of how adaptive leadership and positive psychological resources can together support more cohesive and resilient nursing teams.

### Strengths and limitations of the study

This study had several notable strengths. This is among the first studies to explore the mediating role of psychological capital between chameleon leadership and group cohesion among staff nurses, thereby filling an important gap in nursing leadership research. The study employed a robust methodology, utilizing validated and culturally adapted measurement tools, and advanced statistical analyses, such as path analysis, all of which support the reliability and validity of the findings. Additionally, the relatively large sample size of 282 nurses from a range of departments at a major teaching hospital strengthened the representativeness and applicability of the results across different clinical settings.

While this study provides valuable insights into the relationships among chameleon leadership, psychological capital, and group cohesion, several limitations should be acknowledged. First, the use of a convenience sampling method, rather than random sampling, limits the generalizability of the findings beyond the specific hospital setting studied. The sample may not fully represent the broader nursing population in Egypt or other healthcare systems. Second, cross-sectional design restricts the ability to infer causal relationships between variables. Although associations were identified, longitudinal or experimental studies would be needed to establish directionality or causality. Third, data collected through self-reported questionnaires, which may be subject to response biases such as social desirability or recall bias. Lastly, the study was conducted in a single university-affiliated hospital, which may limit the external validity of the results in different institutional or cultural contexts.

## Conclusion

In conclusion, this study highlights the important mediating role of PsyCap in the relationship between chameleon leadership and group cohesion among staff nurses. The findings indicate that adaptable leadership styles positively influence nurses’ psychological resources, which in turn fosters greater team unity and collaboration. These results underscore the importance of developing leadership flexibility and psychological resilience in healthcare teams [35]. Strengthening these elements could significantly enhance team functioning, job satisfaction, and overall quality of patient care in hospital settings.

### Implications for nursing and health policy

The findings of this study have several implications for nursing practice and health policy. First, leadership development programs should be designed to cultivate chameleon-like adaptability, emotional intelligence, and resilience among nurse managers, preparing them to navigate the complex and rapidly changing healthcare environment better. Second, healthcare institutions should prioritize interventions aimed at enhancing psychological capital among nurses, such as resilience training, optimism workshops, and mentorship programs to promote stronger team cohesion and reduce workforce burnout [36, 37]. Lastly, at the policy level, accrediting and regulatory bodies should integrate leadership adaptability and psychological well-being into their standards for organizational excellence, ensuring that nurse leaders are equipped to foster supportive, cohesive, and high-performance teams.

**Table 1:** Distribution of the studied sample according to demographic characteristics (*n* = 282)

Demographic characteristics	No	%
Age (years)		
< 25	124	44.0
25–34	120	42.6
35–44	27	9.6
≥ 45	11	3.9
Mean ± SD	29.0 ± 7.08	
Gender		
Male	65	23.0
Female	217	77.0
Marital status		
Single	119	42.2
Married	155	55.0
Divorced	6	2.1
Widow	2	0.7
Education level		
Three year deplom	12	4.3
Bachelor of nursing	218	77.3
Postgraduate	52	18.4
Years of experience		
< 5	159	56.4
5–10	88	31.2
> 10	35	12.4
Mean ± SD	5.25 ± 4.96	

**Table 2:** Descriptive analysis of the study variables (*n* = 282)

Variables	Total score	Mean percent score
	Mean ± SD	Mean ± SD
Chameleon Leadership Scale	**35.15 ± 7.55**	**62.87 ± 18.86**
Psychological capital questionnaire	**92.78 ± 13.56**	**71.65 ± 14.12**
Work Self Efficacy	23.29 ± 3.93	72.03 ± 16.38
Optimism	22.25 ± 3.83	67.70 ± 15.97
Hope	4.07 ± 0.81	24.29 ± 4.18
Resilience	22.96 ± 3.99	70.67 ± 16.62
Group Cohesion Scale	**49.59 ± 6.57**	**26.66 ± 6.84**

**Table 3:** Correlation between the studied variables (*n* = 282)

		Chameleon Leadership	Work Self Efficacy	Optimism	Hope	Resilience	Psychological capital
Chameleon Leadership	*r*						
	*p*						
Work Self Efficacy	**r**	0.422*					
	**p**	< 0.001*					
Optimism	**r**	0.427*	0.566*				
	**p**	< 0.001*	< 0.001*				
Hope	**r**	0.469*	0.684*	0.545*			
	**p**	< 0.001*	< 0.001*	< 0.001*			
Resilience	**r**	0.499*	0.608*	0.790*	0.601*		
	**p**	< 0.001*	< 0.001*	< 0.001*	< 0.001*		
**Psychological capital**	**r**	0.535*	0.840*	0.847*	0.838*	0.879*	
	**p**	< 0.001*	< 0.001*	< 0.001*	< 0.001*	< 0.001*	
**Group Cohesion**	**r**	0.511*	0.613*	0.598*	0.612*	0.649*	0.726*
	**p**	< 0.001*	< 0.001*	< 0.001*	< 0.001*	< 0.001*	< 0.001*

r: Pearson correlation coefficient *: Statistically significant at *p* ≤ 0.05

**Table 4:** Multivariate linear regression analysis for factors affecting group cohesion scale (*n* = 282)

Variable	B	Beta	t	*p*	95% CI	
					LL	UL
Chameleon Leadership Scale	0.147	0.169	3.516*	0.001*	0.065	0.230
Psychological capital questionnaire						
Work Self Efficacy	0.338	0.202	3.404*	0.001*	0.142	0.533
Optimism	0.225	0.132	1.959	0.051	-0.001	0.452
Hope	0.293	0.187	3.132*	0.002*	0.109	0.478
Resilience	0.371	0.225	3.112*	0.002*	0.136	0.606

F, p: f and p values for the model. R^2^: Coefficient of determination. B: Unstandardized Coefficients. Beta: Standardized Coefficients

t: t-test of significance. LL: Lower limit. UL: Upper Limit. *: Statistically significant at *p* ≤ 0.05

**Table 5:** Direct and indirect effect

Variable 1		Variable 2	Direct effect	Indirect effect	C.*R*	*p*-value
Work Self Efficacy	←	**Chameleon Leadership**	0.220		7.797*	< 0.001*
Optimism	←	**Chameleon Leadership**	0.217		7.921*	< 0.001*
Hope	←	**Chameleon Leadership**	0.260		8.907*	< 0.001*
Resilience	←	**Chameleon Leadership**	0.264		9.654*	< 0.001*
Group Cohesion	←	**Chameleon Leadership**	0.147	0.097	3.548*	< 0.001*
Group Cohesion	←	**Work Self Efficacy**	0.338		3.434*	< 0.001*
Group Cohesion	←	**Optimism**	0.225		1.977*	0.048*
Group Cohesion	←	**Hope**	0.293		3.161*	0.002*
Group Cohesion	←	**Resilience**	0.371		3.140*	0.002*

Model fit parameters CFI; IFI; RMSEA (1.000; 1.000; 0.089)

CFI = Comparative fit index; IFI = incremental fit index; and RMSEA = Root Mean Square Error of Approximation

Model χ^2^; significance 63.058^*^(< 0.001^*^)

## Data Availability

The data that support the findings of this study are available from the corresponding author upon reasonable request.
